# Study protocol and rationale for a prospective, randomized, double-blind, placebo-controlled study to evaluate the effects of Ashwagandha (*Withania somnifera*) extract on nonrestorative sleep

**DOI:** 10.1097/MD.0000000000011299

**Published:** 2018-06-29

**Authors:** Abhijit Deshpande, Nushafreen Irani, Rathna Balakrishnan

**Affiliations:** International Institute of Sleep Sciences, Thane, Maharashtra, India.

**Keywords:** actigraphy, Ashwagandha, nonrestorative sleep, polysomnography, RSQ-W

## Abstract

**Abstract:**

Nonrestorative sleep (NRS) is one of the cardinal symptoms of insomnia and can occur independent of other components of insomnia. Among the sleep disturbances, NRS has been little studied in the general population, even though this symptom plays an important role in several medical conditions associated with chronic inflammation such as heart disease, fibromyalgia, and chronic fatigue syndrome, as well as various sleep disorders. There is paucity in the literature about effective treatments for NRS. Ashwagandha (*Withania somnifera*) has been demonstrated to reduce anxiety and stress, allowing the body to settle down and prepare for sleep. This study will be a double-blind, randomized, placebo-controlled interventional study in NRS population.

The NRS participants are identified using Restorative Sleep Questionnaire-weekly version (RSQ-W) questionnaire. Actigraphy and polysomnography are used for the objective assessment of sleep. The other assessments used are Hamilton Anxiety Depression Scale (HADS), World Health Organization Quality of Life (WHOQOL) scales, and C-reactive protein. Routine blood and urine analyses will be conducted to assess the safety of treatment. Duration of study for each participant will be 50 days with “day one” for screening followed by randomization for the treatment. The duration for medicine/placebo intake shall be 42 days.

Primary outcome will be to evaluate effect of daily supplement of ashwagandha extract compared with placebo in subjects with NRS at 6 weeks from baseline, as assessed by the total score of RSQ-W.

**CTRI Registration Number:**

CTRI/2017/02/007801

## 1 Introduction

Disturbed sleep is one the commonest encounters of day-to-day life in the fast moving society.^[1,2]^ Most common sleep disturbances are “difficulty in initiating sleep” and “nonrestorative sleep” (NRS). This results in daytime fatigue. In a Swedish survey of 1550 randomly selected subjects from entire Swedish population, almost one-third felt the need for the treatment and yet had not consulted healthcare provider.^[3]^ Insomnia symptoms are highly heterogeneous in terms of causes and manifestations. Recent studies have suggested that NRS symptoms may or may not be present along with insomnia symptoms such as difficulty in initiating and/or maintaining sleep (DIS and/or DMS). Thus, sleep disorders may be grouped as pure DIS and/or DMS, only NRS, and combination of DIS and/or DMS with NRS.^[4]^

NRS can be defined as a moderate to severe complaint of being unrefreshed upon awakening (even if the sleep duration is sufficient according to the subject) occurring at least 3 nights per week during a period of at least 1 month.^[5]^ NRS has been little studied in the general population even though it influences several medical conditions such as heart disease, fibromyalgia, and chronic fatigue syndrome, as well as various sleep disorders. When compared with subjects who have only DIS/DMS, subjects with NRS reported more frequently a variety of daytime impairment (irritability, physical, and mental fatigue) and consulted a physician twice as frequently for their sleeping difficulties.^[5]^ According to International Classification of Sleep Disorders (ICSD), NRS is an associated feature in many intrinsic sleep disorders such as sleep- related breathing disorders and Parasomnias.^[6]^ Other conditions, such as chronic fatigue syndrome and depression, have increased peripheral inflammatory markers and NRS as common features.^[7]^ This underscores the causative relationship between systemic inflammation and NRS.

Currently available treatments for insomnia, including pharmacologic managements and psychological therapy, are designed for DIS and/or DMS rather than NRS, even though NRS is associated with a greater level of functional impairment.^[8]^ Despite recent advances in the development of newer hypnotics in modern medicine, a significant proportion of subjects with sleep disturbances, both locally and internationally, consume herbal hypnotic regularly.^[9]^ Therefore, there is always a search for a candidate molecule for the better management of NRS.

Given the multifactorial involvement in NRS, it seems logical that a medicine that would cause reduction in stress, has antidepressant property, reduces peripheral inflammatory activity, and also help in improving physical performance will be useful in treating NRS.

In multiple clinical trials, Ashwagandha (*Withania somnifera*) has shown to be effective in reduction of chronic inflammation markers such as C-reactive protein (CRP).^[10]^ It was shown to have mild anti-anxiety and anti-depressant effect and effective in reducing postchemotherapy fatigue in breast cancer subjects.^[10–14]^ Thus, Ashwagandha would very likely have an impact on “NRS.” The investigational product studied in this clinical study is an extract of Ashwagandha (*W somnifera*) developed by M/s Arjuna Natural Extracts Ltd., Aluva, Kerala, India, that is standardized to contain about 35% glycowithanolides.

## 2 Methods

### 2.1 Study objectives

The primary objective of this trial will be to evaluate the effect of daily supplementation of Ashwagandha extract compared with placebo in subjects with NRS at 6 weeks from baseline, as assessed by the total score of Restorative Sleep Questionnaire-weekly version (RSQ-W).

The secondary objective will be to compare Ashwagandha and placebo groups on change from baseline up to 6 weeks by actigraphy parameters [average number of awakenings per hour of sleep, average total sleep time (TST) in 1 week, sleep efficiency], nocturnal polysomnogram parameters (only in a subset of 30 subjects) such as sleep onset latency (SOL), wakefulness after sleep onset (WASO), TST, Micro-arousal index and time spent in individual sleep stage, quality of life scores using WHOQOL-BREF scale, depression, and anxiety scores using Hospital Anxiety and Depression Scale and CRP levels.

### 2.2 Study rationale

NRS (with or without DIS and/or DMS) is strongly associated with stress, anxiety, depression, daytime fatigue, and increased CRP. Ashwagandha was shown to be effective in relieving stress and depression. It is also effective in reducing fatigue and inflammatory markers such as CRP levels. Thus, it is proposed that Ashwagandha would likely be effective in reducing NRS.

### 2.3 Study design

This is a prospective, randomized, double-blind, parallel, placebo-controlled, single-center, clinical study. The study will be conducted at International Institute of Sleep Sciences, Thane, Maharashtra, India. The study protocol (AN-02ASH 0816H3-IIS01 version. 01 dated October 18, 2016) is approved by institutional ethics committees and registered with Clinical Trials Registry- India (www.ctri.nic.in) (CTRI registration number: CTRI/2017/02/007801). The study will be conducted according to the Declaration of Helsinki 2013, the ICH-GCP E6 (R1, R2) 1996, and ICMR-National Ethical Guidelines for Biomedical and Health Research, 2006. The investigational product will be Ashwagandha extract 300 mg in size “0” capsule of M/s Arjuna Natural Extracts Ltd., Aluva, Kerala, India, and matching placebo in size “0” capsule for reference group.

A total of 150 subjects will be enrolled in this clinical trial to compare the efficacy and safety of the test versus reference product. Study duration per subject will be 6 weeks. Block-randomization will be performed by the statistician using a computer-generated program with blocks of varying length. The length of blocks will be unknown to study team except the statistician involved in generating the randomization list. The mechanism of implementation of the allocation sequence will be sealed envelope method. Opaque sealed envelopes are prepared based on the randomization list. Each envelope will be opened in sequence just before the randomization of a subject and will be kept under controlled access of statistician. The principal investigator (PI) will take the informed consent before enrolling the subjects and will take all necessary precautions in protecting the confidentiality of the subjects. The subjects will be identified throughout and after the completion of the study only by the allotted randomization number to protect the confidentiality. The PI delegated pharmacist will dispense the study products. As this study is double blinded, the PI and the subject will be blinded toward the interventions. If a need to know arises in which case knowing the identity of interventions is necessary, the PI will be permitted to unblind the subject. Adequate subject enrollment would be achieved by those visiting the site, educational pamphlets, and by study camps conducted in nearby places. The enrolled subjects would be assigned to either of the 2 study groups (75 in each study group). Subjects will be instructed to take 2 capsules of Ashwagandha extract 300 mg or matching placebo once daily in the evening 2 hours before food with water.

### 2.4 Sample size

Differences of 10 points and 15 points for RSQ-W total score are considered as clinically significant and clinically highly significant, respectively. Assuming standard deviation of 25 points, these differences correspond to standardize effect size of 0.4 (small-to-medium) and 0.6 (medium-to-large), respectively, as per Cohen recommendation. The difference of 10 points for RSQ-W total score (range 0–100 points) also translates to about 1-point change on 5-point Likert scale in 4 of 9 items of the RSQ-W. Similarly, the difference of 15 points for RSQ-W total score translates to about 1-point change in 5 of 9 items of the RSQ-W.

Group-sequential method will be used to determine the sample size. The planned sample size of 150 subjects (75 in each study treatment group) will be required to detect a difference of 10 points in change in RSQ-W total score at 6 weeks from the baseline between the study treatment groups, assuming a standard deviation of 25 points and a 15% drop-out rate, to achieve 80% statistical power at 5% (2-sided) level of significance. Two interim analyses are planned to occur after completion of the study of 50 subjects and 150 subjects. If the analysis turns out to have a statistically nonsignificant difference, then the enrollment may extend to 236 subjects. The final analysis will be performed when all 236 subjects complete the study.

### 2.5 Type, sequence, and duration of study periods

This will be a parallel arm treatment study and all the enrolled subjects will be instructed to take study medication for a treatment period of 6 weeks. There will be a total of 8 to 9 visits and/or follow ups: screening, randomization (visit 1, baseline or day 0), follow-up (visit 2 or day 1), follow-up (visit 3 or day 8), follow-up (visit 4 or day 15), follow-up (visit 5 or day 22), follow-up (visit 6 or day 29), follow-up (visit 7 or day 36), follow-up (visit 8 or day 43), and end of study visit (visit 9 or day 50). Laboratory investigation of all the efficacy and safety parameters will be conducted screening and end of the study. Visits 1,3,7,8, and 9 are mandatory for physical presence at investigation site. Visits 4, 5, and 6 can be done via telephone as per subject's convenience. Visit 2 is only for subjects who will be undergoing polysomnography evaluation.

### 2.6 Population

A total of 150 subjects either male or female with age 18 to 65 years will be enrolled in this study. The study eligibility criteria were chosen to maximize generalizability of the results, while taking into account safety and well-being of the participants. The inclusion and exclusion criteria are summarized in Table 1.^[15]^

**Table 1:**
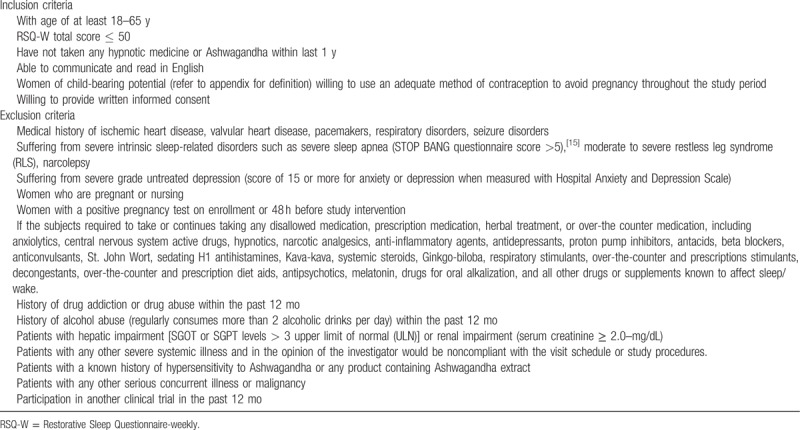
Inclusion and exclusion criteria.

### 2.7 Investigational product and dosage

The investigational product used in this study is a proprietary detoxified root extract of Ashwagandha (*W somnifera*, Solanaceae) and is manufactured by Arjuna Natural Extracts Ltd, Aluva, Kerala, India. Harvested dried roots of Ashwagandha, visually identified by a qualified botanist, are used to manufacture the extract. The extraction solvent used was ethanol: water with a proportion of 70:30 with a raw material to extract ratio of 13:1. The extract was standardized by HPLC to contain 35% glycowithanolides. The analysis of heavy metals was done with ICP-MS and conforms to EU standards (Lead < 0.5 ppm, Arsenic < 1 ppm, Cadmium < 1 ppm, Mercury < 1 ppm). The microbial assay was done using AOAC, BAM method and conforms to EU standards (Yeast/Mould <100 cfu/g, Salmonella Absent/25 g, *Escherichia coli* Absent/10 g). Identically colored and similar sized placebo capsules were used as the placebo.

Ashwagandha extract or matching placebo capsules will be given as two 300 mg capsules once daily in the evening 2 hours before food with water. The dosage of Ashwagandha extract (Shoden) capsules is standardized to contain 21 mg of glycowithanolides per dose. No dose modification will be allowed in the study. Subjects needing concomitant medication will be managed as per the exclusion criteria of the study. Those subjects having adverse events (AEs) that require standard treatments will be discontinued. Investigational product accountability and telephonic follow-up are used to monitor adherence to protocol.

### 2.8 Data monitoring

A Data Monitoring Committee (DMC) will review the safety of the study and the results of the interim analyses and make appropriate recommendations to the trial steering committee. The DMC will be independent from the sponsor and do not have any competing interests. The members have experience in reviewing clinical trial data and have statistical analysis background. Requests for granting public access to the full protocol, participant-level dataset, and statistical code will be as per the SOP of the DMC, which can be sourced with a detailed request to the committee.

### 2.9 Description of any interim analyses and stopping guidelines

Interim analyses for efficacy are planned after 50 and 150 subjects complete the 6-week assessment for the primary outcome (or discontinue the study) using a critical *P* value of ≤.0002 for the first analysis and ≤.012 for the second analysis. These boundaries are obtained using O’Brien–Fleming spending function and based on an overall significance level of 5%. That is, the study is likely to be stopped for strong efficacy signal at the first and second interim analysis if the observed mean difference between the study treatment groups is at least 26 points and 12 points, respectively (assuming SD = 25 points, and drop-out rate = 15%). The study may also be stopped due to futility at any interim analyses on discretion of the DMC. If we do not get a desired statistical difference with the analysis of 150 subjects, then we will continue the enrollment up to 236 subjects and the final analysis will be performed when all 236 subjects complete the study, using a critical *P* value of ≤.0475.

### 2.10 Safety monitoring and adverse events

No AEs have been reported with the use of the product till now in various clinical studies conducted earlier in subjects but as a precautionary measure we are monitoring the routine hematological, biochemical, and urine analysis in the in-house laboratory. In addition, the subjects would be asked an open question like “Have you noticed any change in your health since your last visit?” For all AEs, the investigator will pursue and obtain information about the type, severity, date of onset, treatment given, duration, outcome, causality as assessed by WHO criteria, and decide on whether the AE is serious or not. The serious adverse event (SAE) will be recorded on SAE form and reported to the Ethics Committee and to the Sponsor's representative. The sponsor has taken adequate insurance in case of any need for post trial care or compensation to those who suffer from harm as a result of participation in the trial.

## 3 Measures

Measures include demographic data, vital signs (including heart rate, blood pressure, weight), etc. The primary and secondary study efficacy outcomes are listed in Table 2 and schedule of enrolment, interventions, and assessments is given in Table 3.

**Table 2:**
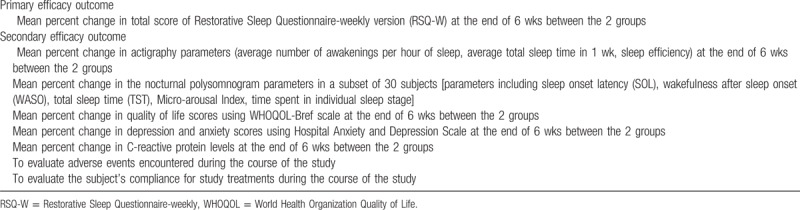
Primary and secondary study efficacy outcome.

**Table 3:**
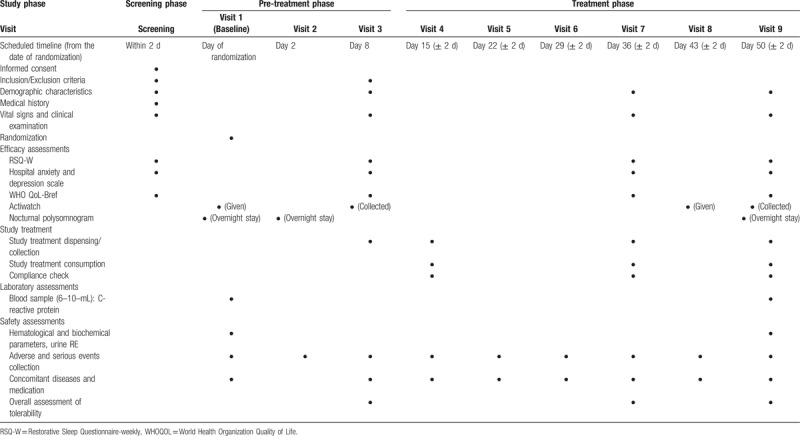
Schedule of enrolment, interventions, and assessments.

### 3.1 Restorative Sleep Questionnaire (RSQ-W) total score

RSQ-W is a validated scale for measuring refreshing quality of sleep. It has 9 items with answers scaled from 1 to 5. Some items are reversed scored. The total score is an average score based on all 9 items and rescaled to a 0 to 100 scale, using the following transformation: RSQ-W Total Score = (RSQ-W average score across completed items – 1) X 25.

### 3.2 WHOQOL-BREF scores

WHOQOL-BREF is a 26-item, self-administered, cross-culturally validated questionnaire in which items are rated on a 5-point scale. The 26 items measure the following broad domains: physical health, psychological health, social relationships, and environment. Domain scores are scaled in a positive direction (i.e., higher scores denote higher quality of life). The mean score of items within each domain is used to calculate the domain score. Mean scores are then multiplied by 4. The second transformation method described in the manual converts domain scores to a 0 to 100 scale. The study will use the English version.

### 3.3 Hospital and Anxiety Scale (HADS) score

Hospital and Anxiety Scale (HADS) is a 14-item cross culturally validated scale with 7 items each in 2 subscales for evaluation of symptoms of anxiety (HADS-A) and depression (HADS-D). It detects symptoms of anxiety and depression, rather than making a diagnosis of the syndrome, and excludes symptoms that may arise from physical illness, insomnia, or fatigue. Each item is scored (0–3) according to severity, with a maximum possible score of 21 for each subscale. A score of 0 to 7 indicates no anxiety or depression, a score of 8 to 10 indicates a borderline case, and a score of 11+ indicates presence of anxiety and/or depression. The study will be using English version for the study.

### 3.4 Actigraphy parameters

Actigraphy is a noninvasive method of monitoring human rest/activity cycles. It is validated against gold standard Nocturnal Polysomnogram. Using software algorithms, sleep parameters such as latency to sleep, sleep duration, awakenings per night, and sleep efficiency are calculated. We will be able to distinguish between 3 groups (pure NIS, pure NRS, and a mixture of both) using actigraphy. Another advantage of actigraphy will be assessment of sleep in subject's own natural environment.

### 3.5 Nocturnal polysomnography (NPSG) parameters

Nocturnal polysomnography (NPSG) is considered a gold standard test for objective measurements of sleep parameters such as total bed time, TST, sleep efficiency, SOL, WASO, micro arousal index, % of each sleep stage (NREM stage 1, 2, and 3 and REM), apnea/hypopnea index (AHI), and periodic limb movement index (PLMI). The NPSG monitors many body functions, including brain (EEG), eye movements (EOG), muscle activity or skeletal muscle activation (EMG), and heart rhythm (ECG) during sleep. Recordings will be taken from bedtime to awakening in the morning and continuous audiovisual infra red monitoring of the subjects. Sleep stages shall be manually scored according to guidelines in American Academy of Sleep Medicine (AASM) Manual for the Scoring of sleep and Associated events in 30-second epoch page.

### 3.6 Serum C-reactive protein levels (CRP)

Serum CRP, a systemic marker of inflammation, is associated with an increased risk for a host of chronic diseases. Reduction in the CRP level would suggest reduction in chronic inflammation.

### 3.7 Statistical analysis

The data will be collected from paper case report forms (manually counter checked with source files by the data entry personnel) by the investigator and given to the statistician who will analyze the data for demographics, efficacy, and safety. Data will be presented as mean ± SD/SE or number (percentage). Descriptive statistics will be used for different variables at baseline. *P* value of < .05 will be considered as statistically significant. Standard statistical tests will be used to analyze the data obtained. For efficacy analysis both, modified intention-to-treat (mITT) analysis and per-protocol (PP) analysis will be done. The PP analysis will be considered as definitive, while the mITT analysis will be considered as supportive during the trial analysis.

## 4 Discussion

The quality of sleep is one of the major hurdles that sleep therapists face in today's society. NRS is one of the indicators of poor sleep quality that results in day time fatigue and lethargy.

Ashwagandha (*W somnifera),* also commonly known as Indian ginseng, is a shrub plant belonging to the Solanaceae family. The various plant parts of (e.g., roots, leaves, and berries) of Ashwagandha have been used by traditional Indian Ayurvedic medicine for thousands of years to help with inflammation, sexual issues, nerve tissue damage, stress, anxiety, insomnia, and many other ailments. The major phytochemical constituents reported from Ashwagandha are alkaloids (isopelletierine, anaferine, cuscohygrine, anahygrine, tropine, somnine, sominiferine, withanine, etc), steroidal lactones (withanolides, withaferins), saponins containing an additional acyl group (sitoindoside VII and VIII), withanolides with a glucose at carbon 27 also known as glycol-withanolides (sitoindoside IX and X), and amino acids such as aspartic acid, proline, tyrosine, alanine, glycine, etc.^[16]^ A lot of herbal supplements are commercially available containing majority of alkaloids and small amount of withaferine. The pharmacological activity of such products has been claimed as due to presence of alkaloids and/or withaferine. European Food Safety Authority (EFSA) has categorized some of the alkaloids of Ashwagandha as toxic and harmful.^[17]^ These include anaferine, anahygrine, withanine, sominiferine, somnine, tropine, etc. Apart from alkaloids, withaferine A has also been categorized as cytotoxic lactones by EFSA.^[18]^

The Ashwagandha extract developed by M/s Arjuna Natural Extracts Ltd., Aluva, Kerala, India, removes all of the alkaloids, thus minimizing the toxic and harmful elements and is standardized to contain 35% glycowithanolides.

Ashwagandha's anti-anxiety effects have been evaluated in published literature.^[11]^ It also shows promise for relieving insomnia and stress-induced depression.^[12]^ Ashwagandha can significantly reduce cortisol concentrations and the immunosuppressive effect of stress.^[13]^ It also has shown to have a lowering effect on CRP denoting reduction in pro-inflammatory responses in body.^[10]^ Beyond reducing stress levels, Ashwagandha can improve physical performance and reduce fatigue sensation in both sedentary people and athletes.^[17]^ In fact, Ashwagandha has been shown to improve fatigue score in post-chemotherapy breast cancer subjects.^[14]^

The inclusion criteria were kept broad and the exclusion criteria were considered to protect study participants and increase the chance of finding a treatment effect. The protocol amendments will only be incorporated in mutual agreement between the sponsor and the investigator. The amendments will be submitted to the ethics committee for its approval.

All subjects will be evaluated in their own setting with Actigraphy, thus giving opportunity to examine the changes in real-life scenario. The subjects will be instructed to wear the Actiwatch continuously for a period of 7 days. The noncompliance could be objectively detected by the software algorithms incorporated in the device.

Although compliance will not be formally measured in this study, a regular phone call as well as personal visits to the institute at regular intervals shall be done. Dosage format will be once a day in the evening, which is practical and relevant to the real-life scenario.

The analyses will include all participants regardless of their compliance but will not include dropouts. We will also assess the heterogeneity of treatment effect in several relevant subgroups. The trial results will be communicated through publication in leading journals.

The sample size was calculated to give enough power for study without resorting to crossover design thus avoiding potential chance for certain errors such as carryover effect of the medicine.

The motivation for the trial design was based on the very common occurrence of nonrefreshing sleep (NRS) in society, its resultant, and the lack of high-quality evidence for the efficacy of the commonly used medication. It is anticipated that this study will result in evidence-based treatment of subjects suffering from nonrefreshing sleep. If negative, the results of this study will still be critical for further efforts to elucidate epidemiology, pathophysiology, and new treatment targets for NRS.

## 5 Conclusion

NRS is relatively common and yet underexplored issue. There is a paucity of knowledge about its prevalence, clinical implications, and treatment. This study will be the first of epidemiological studies to explore the prevalence of this entity in Indian population. To our knowledge, this will be the first study to explore objective correlates of NRS using actigraphy, which gives us the advantage of measuring these in subject's own environment. Ashwagandha has been shown to be a useful supplement in many conditions such as postchemotherapy fatigue in double-blind, randomized studies. This study will also be the first to explore specific changes in sleep quality.

This study will be limited to educated people who can read and/or understand English. Although an argument can be made in defense of this limitation, NRS has shown to be more prevalent in educated, younger demographic. Due to financial constraints, NPSG will not be performed in all populations.

## Acknowledgments

The authors deeply acknowledge the gracious help of M/s Arjuna Natural Extracts Ltd., Aluva, Kerala, India, for providing Ashwagandha extract capsule and financial assistance to conduct the study.

## Author contributions

**Conceptualization:** Abhijit Deshpande, Nushafreen Irani, Rathna Balakrishnan.

**Data curation:** Abhijit Deshpande, Nushafreen Irani.

**Formal analysis:** Abhijit Deshpande.

**Funding acquisition:** Abhijit Deshpande.

**Investigation:** Abhijit Deshpande, Nushafreen Irani.

**Methodology:** Abhijit Deshpande, Rathna Balakrishnan.

**Project administration:** Nushafreen Irani, Rathna Balakrishnan.

**Resources:** Abhijit Deshpande.

**Software:** Abhijit Deshpande.

**Supervision:** Abhijit Deshpande, Nushafreen Irani, Rathna Balakrishnan.

**Validation:** Abhijit Deshpande.

**Visualization:** Abhijit Deshpande.

**Writing – original draft:** Abhijit Deshpande.

**Writing – review & editing:** Abhijit Deshpande, Nushafreen Irani, Rathna Balakrishnan.
